# Prenatal Phthalate Exposure and Anogenital Distance in Male Infants

**DOI:** 10.1289/ehp.114-a88b

**Published:** 2006-02

**Authors:** Shanna H. Swan

**Affiliations:** Department of Obstetrics and Gynecology, University of Rochester, School of Medicine and Dentistry, Rochester, New York, E-mail: shanna_swan@urmc.rochester.edu

In our article “Decrease in Anogenital Distance among Male Infants with Prenatal Phthalate Exposure” (Swan et al. 2005), we reported results of our recent study on the relationship between anogenital distance (AGD) in boys and their mother’s urinary concentration of phthalate metabolites (Swan et al. 2005).

The primary question we addressed was the relationship between the concentration of phthalate metabolites in maternal prenatal urine and the AGD, or the more appropriate derived measure anogenital index (AGI = AGD/weight), in human male offspring. We designed our study to focus on this specific measurement because of highly reliable results in the animal literature showing that certain phthalates reduce AGD (and AGI) in rodents and because, as continuous variables, AGD and AGI would not require a large sample size to demonstrate this relationship, if it existed. Changes in the frequency of a dichotomous and relatively rare end point such as frank cryptorchidism, also caused in animals by prenatal phthalate exposure, require far larger sample sizes. Secondarily, we looked at AGI in relation to other genital measurements (penile volume, testicular descent, and scrotal size), examining these interrelationships in several ways.

In our article (Swan et al. 2005), we reported that urinary concentrations of four phthalate metabolites [mono-*n*-butyl phthalate (MBP), monobenzyl phthalate (MBzP), monoethyl phthalate (MEP), and mono-isobutyl phthalate (MiBP)] were inversely and significantly related to AGI. We also examined three metabolites of diethylhexyl phthalate (DEHP). Although the associations between AGD and the secondary DEHP metabolites [mono-2-ethyl-5-oxohexyl phthalate (MEOHP) and mono-2-ethyl-5-hydroxyhexyl phthalate (MEHHP)] were suggestive, they were not statistically significant, and the metabolite MEHP appeared to be unrelated to AGI.

We examined the relationship between AGI and testicular descent in several ways, varying whether each of these variables was entered into the analysis untransformed (e.g., as they were recorded in the examination) or as dichotomous variables.

AGD was measured by the examiner using a Vernier calipers. Both AGD and AGI are continuous, and approximately normally distributed, variables. The degree of descent of each testicle was categorized as follows: 0 = normal, 1 = normal retractile, 2 = high scrotal, 3 = suprascrotal, 4 = inguinal, and 5 = nonpalpable or ectopic. The testicular placement score (TPS) is the sum of the recorded value for the left and right testicle. Therefore, the lower the TPS, the more complete the testicular descent. We first examined the relationship between AGI and testicular descent by calculating the correlation coefficient between AGI and TPS (an ordinal variable). In the complete data set, including 134 boys with genital examination, AGI is significantly and inversely related to TPS (correlation coefficient −0.201, *p*-value 0.021). That is, shorter AGI was significantly associated with less complete testicular descent.

This analysis assumes that TPS is an interval variable; for example, the difference between a score of 0 (both testicles “normal”) and 1 (one testicle “normal” and one “normal retractile”) is equal to that between 1 and 2 (either both “normal retractile,” or one “normal” and one “high scrotal”). We also examined TPS as a dichotomous variable, which does not require this assumption. For this purpose, testicular descent was coded as 0 and called “complete” if both testicles were rated as either normal or normal retractile; otherwise, it was coded as 1 and called “incomplete.” This dichotomous variable was also significantly correlated with AGI (correlation coefficient −0.192, *p*-value 0.027). Since results by these two methods were similar, we did not include this latter analysis in our article (Swan et al. 2005).

As is common practice in epidemiologic analyses, we also dichotomized AGI to create two groups to serve as cases and controls. For this purpose, we classified boys into “short” AGI (< 25% of expected for age) or “not short.” We looked at the proportion of boys with incomplete testicular descent in these groups. We incorrectly stated the *p*-value for not short (0.136) in our article (Swan et al. 2005) to be statistically significant. That this analysis was not statistically significant, while the analysis of the AGI as continuous variable was, is not surprising; dichotomizing a continuous variable results in a loss of power, and thus a larger sample size is needed to achieve a similar level of statistical significance (Ragland 2002).

In conclusion, in all analyses boys with shorter AGI had less complete testicular descent, and significantly so for the two analyses in which AGI was treated as a continuous variable. The miscalculation of statistical significance for one analysis, while unfortunate, in no way alters any of our conclusions. Also, this error does not weaken this article’s (Swan et al. 2005) support for the importance of examining patterns of subtle changes in humans, as suggested by toxicology, when assessing the effects of environmental exposures.
